# Editorial: Physiological Aspects of Non-proteinogenic Amino Acids in Plants

**DOI:** 10.3389/fpls.2020.519464

**Published:** 2020-12-17

**Authors:** Georg Jander, Uener Kolukisaoglu, Mark Stahl, Gyeong Mee Yoon

**Affiliations:** ^1^Boyce Thompson Institute, Ithaca, NY, United States; ^2^Center for Plant Molecular Biology, University of Tuebingen, Tuebingen, Germany; ^3^Department of Botany and Plant Pathology, Center for Plant Biology, Purdue University, West Lafayette, IN, United States

**Keywords:** non-proteinogenic, amino acid, metabolism, biosynthesis, plant

In addition to the canonical 20 amino acids that constitute the essential building blocks of proteins, plants produce a wide variety of non-proteinogenic amino acids (NPAAs; Fowden, 1981, Rosenthal, 1982, Barrett, 1985, Bell, 2003). Some of these plant metabolites are components of central metabolism, serving as intermediates in biosynthetic pathways or as signaling molecules during plant stress responses. NPAAs such as ornithine, citrulline, arginosuccinate, homoserine, homocysteine, and cystathionine, are well-studied metabolic intermediates and are likely to be present in all plant species. Other commonly encountered plant NPAAs, for instance pipecolic acid with its derivatives, can function as signaling molecules that influence plant development, physiology, and defense responses (Huang et al., 2020).

A particularly noteworthy NPAA, γ-aminobutyric acid (GABA), is essential for many physiological and developmental processes in plants, including energy dissipation, maintenance of carbon/nitrogen balance, pollen tube growth, and fruit development (Kinnersley and Turano, 2000, Palanivelu et al., 2003, Fait et al., 2008, Snowden et al., 2015, Amir et al., 2018). Functioning as both signaling molecule and a regulator of plant metabolism, GABA can modulate plant immune responses (Kim et al., 2013, Wang et al., 2019, Deng et al., 2020, Tarkowski et al., 2020). Numerous studies have shown a role for GABA accumulation in protecting plants against abiotic stresses such as drought and salinity (Bor et al., 2009, Akcay et al., 2012, Vijayakumari and Puthur, 2015, Mekonnen, 2017, Carillo, 2018, Rezaei-Chiyaneh et al., 2018, Jin et al., 2019, Podlesakova et al., 2019).

NPAAs that are not part of primary metabolism are often defense-related, providing protection against pests and pathogens, and typically have a more sporadic distribution in the plant kingdom (Bell, 1976). For instance, many legumes accumulate large amounts of canavanine or other NPAAs that not only function as defensive metabolites but also serve for nitrogen storage in the seeds (Huang et al., 2011). Canavanine is a structural analog of arginine and exerts its toxicity in animals by interfering with arginine-related metabolism, including nitric oxide synthase and incorporation of arginine into proteins (Bence and Crooks, 2003). In new research on the toxicity of canavanine in plants, Staszek et al. show that the canavanine-mediated inhibition of nitric oxide biosynthesis leads to formation of differentially nitrated proteins and a disruption of the antioxidant system in tomato roots.

Another NPAA, 1-aminocyclopropane carboxylate (ACC), is the direct precursor of ethylene, a gaseous hormone regulating a wide ranges of developmental and stress-related processes in plants (e.g., Lee et al., 2019, Seo and Yoon, 2019). However, as discussed by Polko and Kieber, ACC itself also functions as a plant signaling molecule. Physiological processes in plants that are influenced directly by ACC include stomatal development, cell wall biosynthesis, stress responses, and pathogen interactions (Xu et al., 2008, Tsuchisaka et al., 2009, Tsang et al., 2011, Yin et al., 2019). The levels of ACC in plants are critical for ethylene production and seem to be influenced by another group of NPAAs, the *D-*Amino acids. *D-*Amino acid isomers of the proteinogenic *L*-amino acids are produced by soil microbes and are taken up by plant roots, but can also be produced by plants themselves (Genchi, 2017). Although some *D*-amino acids are toxic to *Arabidopsis thaliana* (Arabidopsis) at low concentrations (Erikson et al., 2004), the metabolism of *D-*amino acids strongly varies between different Arabidopsis ecotypes (Gordes et al., 2013). Suarez et al. used natural accessions and transgenic mutant lines to identify and investigate AtDAT1, a major *D-*amino acid transaminase in Arabidopsis. Decreased activity of this enzyme leads to enhanced susceptibility to *D-*methionine and increased *D-*amino acid abundance stimulated accumulation of ethylene. In this study it was demonstrated, that the regulation of *D-*methionine and ACC derivatives in plants are interlinked. However, the detailed mechanisms by which *D-*amino acids induce ethylene production remain to be investigated.

β-Amino acids, which have the amino group attached to the β-carbon rather than the adjacent α-carbon, have been reported in many plant species (Kudo et al., 2014). Whereas, some β-amino acids, for instance β-tyrosine, have likely defensive functions in plants (Yan et al., 2015), others are essential components of primary metabolism. Parthasarathy et al. review the biosynthesis and function of β-alanine, which is not only a component of vitamin B_5_ and thereby is essential for Coenzyme A function, but also contributes to plant responses to both biotic and abiotic stresses. Although the β-alanine biosynthetic pathways are not yet completely elucidated in plants, spermine, spermidine, propionate, and uracil are known metabolic precursors.

The biosynthetic pathways of proteinogenic amino acids, and by extension the biosynthesis of NPAAs that serve as intermediates in these pathways, have been elucidated in Arabidopsis and other plant species (Jander and Joshi, 2010). However, the biosynthetic pathways and/or metabolic functions have been unraveled for only a few of the hundreds of other plant NPAAs, including *D-*amino acids, β-amino acids, other isomers, and structural mimics. Thus, there are many opportunities for novel discoveries in this research area. In particular, with the development of new research methods for studying non-model plant species at the molecular level, it will be possible to study the biosynthesis pathways, as well as structural, defensive, and signaling functions, of NPAAs that are not present in Arabidopsis.

## Author Contributions

GJ wrote the first draft. All authors contributed revisions and approved the published version of the manuscript.

## Conflict of Interest

The authors declare that the research was conducted in the absence of any commercial or financial relationships that could be construed as a potential conflict of interest.
